# Pou5f1/Oct4 Promotes Cell Survival via Direct Activation of *mych* Expression during Zebrafish Gastrulation

**DOI:** 10.1371/journal.pone.0092356

**Published:** 2014-03-18

**Authors:** Kay Kotkamp, Esther Kur, Björn Wendik, Bożena K. Polok, Shifra Ben-Dor, Daria Onichtchouk, Wolfgang Driever

**Affiliations:** 1 Developmental Biology, Institute Biology I, Faculty of Biology, Albert-Ludwigs-University Freiburg, Freiburg, Germany; 2 Biological Services, Weizmann Institute of Science, Rehovot, Israel; 3 BIOSS - Centre for Biological Signalling Studies, Freiburg, Germany; University of Birmingham, United Kingdom

## Abstract

Myc proteins control cell proliferation, cell cycle progression, and apoptosis, and play important roles in cancer as well in establishment of pluripotency. Here we investigated the control of *myc* gene expression by the Pou5f1/Oct4 pluripotency factor in the early zebrafish embryo. We analyzed the expression of all known zebrafish Myc family members, *myca*, *mycb*, *mych*, *mycl1a, mycl1b*, and *mycn*, by whole mount *in situ* hybridization during blastula and gastrula stages in wildtype and maternal plus zygotic *pou5f1* mutant (MZ*spg*) embryos, as well as by quantitative PCR and in time series microarray data. We found that the broad blastula and gastrula stage *mych* expression, as well as late gastrula stage *mycl1b* expression, both depend on Pou5f1 activity. We analyzed ChIP-Seq data and found that both Pou5f1 and Sox2 bind to *mych* and *mycl1b* control regions. The regulation of *mych* by Pou5f1 appears to be direct transcriptional activation, as overexpression of a Pou5f1 activator fusion protein in MZ*spg* embryos induced strong *mych* expression even when translation of zygotically expressed mRNAs was suppressed. We further showed that MZ*spg* embryos develop enhanced apoptosis already during early gastrula stages, when apoptosis was not be detected in wildtype embryos. However, Mych knockdown alone did not induce early apoptosis, suggesting potentially redundant action of several early expressed *myc* genes, or combination of several pathways affected in MZ*spg.* Experimental *mych* overexpression in MZ*spg* embryos did significantly, but not completely suppress the apoptosis phenotype. Similarly, p53 knockdown only partially suppressed apoptosis in MZ*spg* gastrula embryos. However, combined knockdown of p53 and overexpression of Mych completely rescued the MZ*spg* apoptosis phenotype. These results reveal that Mych has anti-apoptotic activity in the early zebrafish embryo, and that p53-dependent and Myc pathways are likely to act in parallel to control apoptosis at these stages.

## Introduction

Apoptosis plays a crucial role during development and maintenance of homeostasis in multicellular organisms by eliminating damaged or unneeded cells [1], [2]. Programmed cell death is intensely studied in model organisms, because its deregulation is involved in many diseases including cancer, Alzheimer's disease, or immune deficiencies [3], [4]. Today many components of the apoptosis pathway and their regulatory interactions are known [5]–[7]. In recent years, zebrafish have been increasingly used as model for studying apoptosis, because of the ease of experimental manipulation and the close homology of the apoptosis pathway core factors between fish and mammals [8]–[13].

Myc proteins are transcription factors involved in regulation of various cellular functions including cell-cycle progression [14], differentiation [15], [16], cell growth [17], and apoptosis [18]. Myc forms heterodimers with its transcriptional co-regulator Max to bind specific DNA sites in the promoter regions of its target genes [19], [20]. It was shown that Myc might be associated both with gene activation [21], [22] and repression [23], [24]. The *myc* proto-oncogene family consists of the three members c-*myc* [25], N-*myc* [26] and L-*myc* [27]. Myc deregulation is often linked to tumor formation in animals and human [28]–[30]. During normal mouse embryonic development c-*myc* is expressed in many different cell types [31]–[34]. C-Myc was shown to correlate with cell proliferation in several tissues, while it induces or sensitizes cells to apoptosis in others [35]. L-*myc* and N-*myc* expression are restricted to specific tissues in the course of cell differentiation [31]–[34]. The importance of c-Myc in promoting early development was shown by the fact that c-*myc* homozygous knock-out mice are embryonic lethal [36], and its role in stem cell pluripotency when mouse fibroblasts were reprogrammed by overexpressing c-Myc together with Oct4, Sox2 and Klf4 [37]. In embryonic stem cells, Myc makes complex contributions to pluripotency [38]–[40].

In zebrafish, homologous genes for all *myc* family members have been identified [41], [42]. As a result of the genome duplication that occurred in the evolution of teleosts [43] two fish paralogues each exist for c-*myc* (*myca* and *mycb*) and L-*myc* (*mycl1a* and *mycl1b*; Figure S1)[41], [42]. In contrast, there is only one homologous gene for N-*myc* known in zebrafish, *mycn* [42], [44]. Recently, a new member of the *myc* gene family, *mych*, was identified in zebrafish. Mych shows a high similarity to N-Myc and c-Myc in its C-terminal amino acid sequence, but has no direct orthologous gene in higher vertebrates [41], [45]. Zebrafish *myc* homologous genes are differentially expressed during embryonic development, and in specific adult tissues [41], [42], [44], [45]. The spatial distributions of the early gene expression patterns have been previously described for *mych*, *mycn* and *mycl1a*. All three genes show a broad expression at blastula and gastrula stages [44]–[46]. Meijer et al. [41] described the postgastrula expression patterns of all known zebrafish *myc* homologues in great detail (see also Figure S2). However, the functions of *myc* genes in early zebrafish development are poorly understood. The recently identified *mych* gene has been linked to cell survival and neural crest development [45].

In mammalian ES cells, *myc* genes are regulated by the master stem cell factors Pou5f1 (Oct4) and Nanog [38], [40]. Previous work in our lab revealed that the transcript levels of two zebrafish *myc* genes, *mycl1b* and *mych*, are reduced in *pou5f1* (also named Pou2 or Pou5f3; www.zfin.org) maternal and zygotic (MZ*spg*) mutant embryos as judged from microarray analysis [47]. Here we analyzed the early expression patterns of the zebrafish *myc* genes and their expression in Pou5f1 deficient embryos. We showed that the zebrafish *mych* and *mycl1b* genes are bound by Pou5f1 and are likely direct targets of Pou5f1. We further showed that MZ*spg* gastrulae display enhanced apoptosis compared to wildtype (WT) controls. The apoptosis phenotype was partially rescued by *mych* overexpression, indicating a role of Mych in regulation of cell survival during gastrulation.

## Results

### Pou5f1 activity is required to induce zygotic expression of *mych* and *mycl1b*

Temporal expression profiles from time series microarray analyses (zygote to 8 hours post fertilization - hpf) of wildtype (WT) and Pou5f1 maternal and zygotic deficient embryos (MZ*spg*) [47] have revealed *myc* genes potentially regulated by Pou5f1 in the early zebrafish embryo (Figure S3). For the zebrafish c-*myc* homologues *myca* and *mycb*, in MZ*spg* embryos compared to WT, the mRNA levels derived from maternal expression have been found to be reduced before onset of zygotic transcription (midblastula transition - MBT), but elevated at post-MBT stages (Figure S3A and C). *mycl1a* expression was not altered in Pou5f1 mutants compared to WT (Figure S3E). *mycl1b* expression in MZ*spg* has been found to be normal during blastula and early gastrula stages, but downregulated during late gastrulation (Figure S3G). The expression of *mych* and *mycn* has been shown to be strongly activated after MBT in WT embryos, but not in Pou5f1 mutants (Figure S3I and K). Whereas the induction of *mycn* expression only appeared to be delayed in MZ*spg* embryos (Figure S3I), *mych* continued to be expressed at strongly reduced levels in MZ*spg* throughout gastrulation (Figure S3K). These findings suggested that Pou5f1 function is crucial for a proper regulation of *mycl1b* and *mych* gene expression in early zebrafish development.

**Figure 1:**
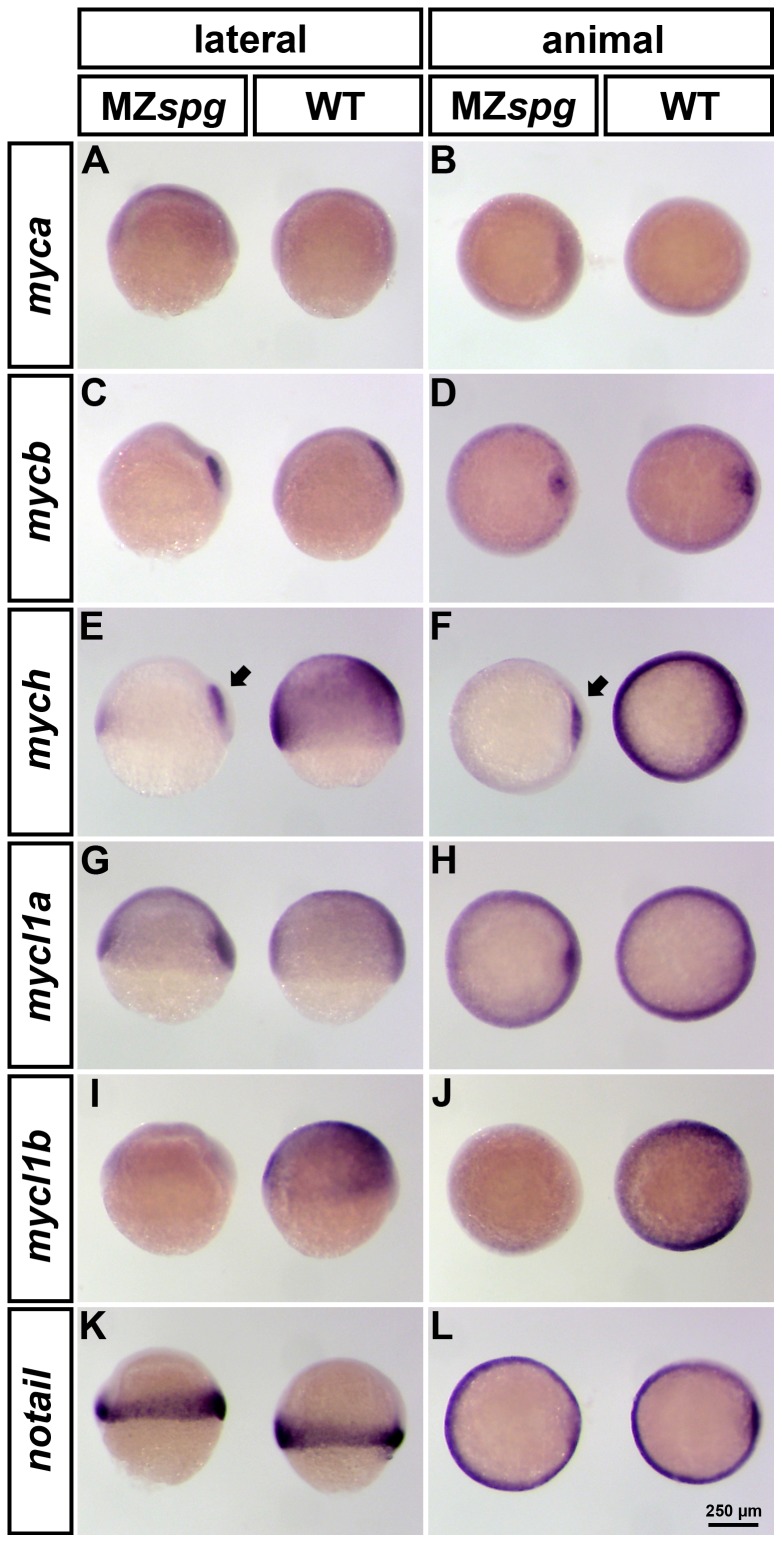
Spatial expression pattern of zebrafish *myc* genes in WT and MZ*spg* embryos at 60% epiboly. Whole mount *in situ* hybridization (WISH) analysis of *myca*, *mycb*, *mych*, *mycl1a* and *mycl1b* expression in WT (right embryo in each panel) and MZ*spg* (left embryo in each panel). All embryos are shown in lateral (left column) and animal (right column) views with dorsal oriented to the right. All analyzed *myc* genes are broadly expressed in mid-gastrula embryos, except for c-*myc* homologues, *myca* and *mycb*. *mycb* is specifically expressed in the shield, whereas *myca* was not detectable at this stage (A-D). *mych* and *mycl1a* in addition have a strong expression domain in the involuting axial mesoderm (E-H). Only *mych* (E-F) and *mycl1b* (I-J) depend on the function of Pou5f1 and their expression is strongly decreased in MZ*spg* mutants. However, the *mych* expression domain in the involuting axial mesoderm is less affected in Pou5f1 deficient embryos (E-F; arrows). We used *notail* as control, because its expression is not altered in MZ*spg* mutants compared to WT (K-L).

To confirm the microarray data and to obtain more information about the spatial distribution of gastrula stage *myc* gene expression patterns and their changes in MZ*spg* embryos, we performed whole-mount *in situ* hybridization (WISH) expression analysis for *myca*, *mycb*, *mych*, *mycl1a* and *mycl1b* in WT and MZ*spg* mutants at 60% epiboly (Figure 1). Early expression patterns of *mych* and *mycn* have been previously reported [44], [45]. The mesodermal marker *no tail* (*ntl*; [48] was used as a control for this experiment, because *ntl* is not regulated by Pou5f1 [47]. Only one of the two c-*myc* homologs, *mycb*, was detected with spatially restricted expression at mid-gastrulation (Figure 1A-D). At 60% epiboly *mycb* was specifically expressed in the region of the embryonic shield (Figure 1C-D). The expression patterns of both *myca* and *mycb* displayed no alterations in Pou5f1 deficient embryos compared to WT. The slightly higher microarray expression levels of *myca* and *mycb* at this stage in MZ*spg* (Fig S3A,C) were not clearly detected by WISH except for a minor increase in stain intensity in MZ*spg*. *mych*, *mycl1a* and *mycl1b* were found to be broadly expressed throughout the blastoderm at 60%-epiboly, while *mycl1b,* in contrast to *mych* and *mycl1a,* was excluded from the embryonic shield (Figure 1E-J). We found that in MZ*spg* mutants *mych* and *mycl1b* expression were strongly downregulated (Figure 1E-F and I-J). Therefore, *mych* and *mycl1b* expression appeared to depend on the Pou5f1 transcription factor. Interestingly, expression of *mych* was found to depend on Pou5f1 activity in most early gastrula cells, except for the cells of the embryonic shield (Figure 1E-F).

**Figure 2:**
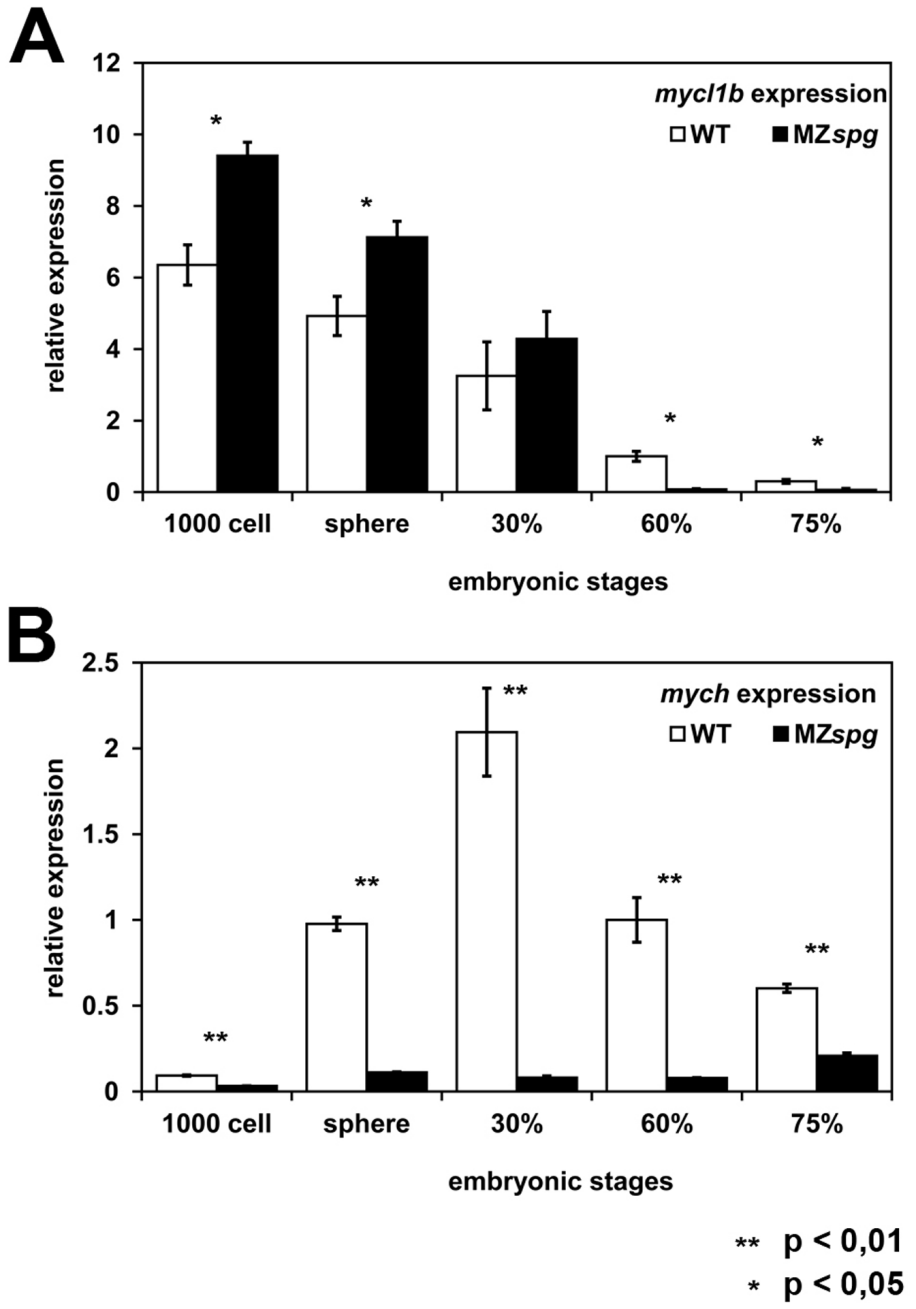
Temporal analysis of *mych* and *mycl1b* expression in WT and MZ*spg*. Relative expression levels of *mych* and *mycl1b* in WT and MZ*spg* embryos for five distinct time points, between 1000-cell (MBT) and 75%-epiboly stage using quantitative real-time PCR. Bars show average values of three independent biological replicates each. The WT expression at 60%-epiboly stage was normalized to 1 for both genes. *mycl1b* is maternally expressed and declines as embryonic development progresses (A, white bars). In MZ*spg* mutants, *mycl1b* expression is upregulated at MBT, likely reflecting higher maternal mRNA levels, but in comparison to WT it declines faster during gastrulation (A, black bars). In WT *mych* is activated at MBT, than its expression increases until 30%-epiboly before it starts to slowly fade (B, white bars). *mych* expression levels are up to 26-fold reduced in MZ*spg* mutants compared to WT during early embryogenesis (B, black bars).

We next quantified the expression of *mych* and *mycl1b* in WT and MZ*spg* embryos for five different developmental stages ranging from 1000-cell (MBT) to 75% epiboly by relative quantitative real-time PCR (qPCR) (Figure 2). We used the housekeeping gene *translation elongation factor 1α* (*ef1α* also named *eef1a1l1*) as reference gene to normalize the expression levels of *mycl1b* and *mych* in MZ*spg* and WT. The developmental expression profiles, determined by qPCR, for both genes were in agreement with microarray results (Figure S3G,K) [47]. The developmental profile for *mycl1b* showed a strong signal at mid-blastula transition in WT and Pou5f1 mutants and expression decreased during gastrulation (Figure 2A). This suggested that most of the early *mycl1b* mRNA is maternally expressed. In comparison to WT, the expression level of *mycl1b* in MZ*spg* was about 1.5 times higher at MBT, but found to decline rapidly in MZ*spg* mutant embryos during gastrulation, resulting in a 12.5 fold downregulation compared to WT at 60%-epiboly (Figure 2A). This difference may be caused by two mechanisms: Pou5f1 may protect maternal *mycl1b* by direct or indirect mechanisms, or Pou5f1 may induce zygotic expression of *mycl1b*. In contrast to *mycl1b*, *mych* was not detected to be expressed maternally. *mych* expression was found to be activated immediately after MBT in WT embryos and to reach a high point at 30% epiboly before declining again (Figure 2B). In MZ*spg* embryos *mych* expression was strongly reduced at all analyzed developmental stages (Figure 2B). Our results demonstrate that the Pou5f1 transcription factor is important for the early zygotic activation of *mych* expression, and for maintaining *mycl1b* RNA levels. In summary, our data indicate that Pou5f1 function is important for the activation of the early zygotic expression of *mych*, and *mycl1b* during the first 8 hours of development.

### *mych* and *mycl1b* are directly activated by Pou5f1

To distinguish between direct and indirect Pou5f1 targets, Onichtchouk et al. [47] performed *pou5f1* mRNA overexpression experiments in MZspg embryos, and inhibited translation of zygotic mRNAs after MBT by adding the translation elongation inhibitor cycloheximide (CHX) at the 64-cell stage. They compared by microarray analysis expression profiles at 30% epiboly of embryos injected with *pou5f1* mRNA versus non-injected controls, both treated with CHX, such that only the expression of direct Pou5f1 targets should be differentially affected. We analyzed these published data to answer the question whether the *myc* genes may be regulated directly or indirectly by Pou5f1 (Figure S3). We found that expression of *mych*, *mycl1b,* and the *c-myc* homologous genes were more than 2-fold increased after the injection of *pou5f1* mRNA and subsequent addition of CHX, which suggests direct regulation by Pou5f1 (Figure S3B, D, H and L).

**Figure 3:**
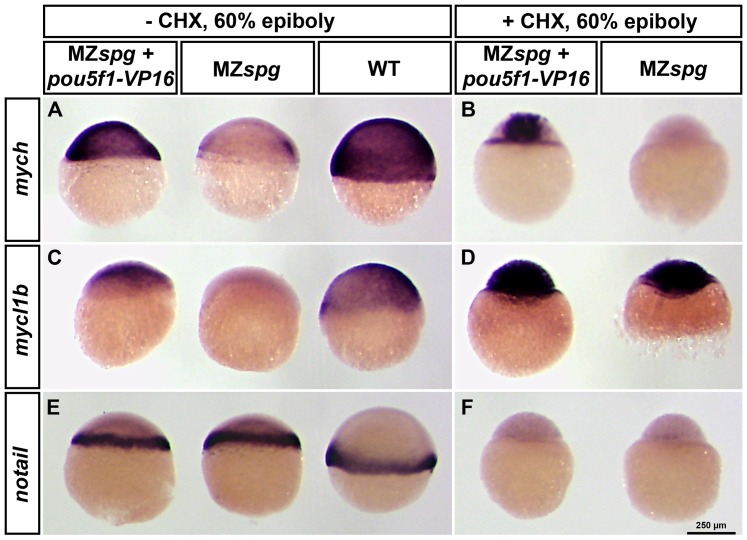
*mych* and potentially also *mycl1b* are directly regulated by Pou5f1. Pou5f1 overexpression in MZ*spg* mutants by *pou5f1-VP16* mRNA injection into one-cell stage embryos (A-F; left embryos in each panel) and non-injected controls (A, C and E; MZ*spg* middle and WT right embryos in each panel; B, D and F; MZ*spg* right embryo in each panel). The embryos in B, D and F are in addition treated with CHX starting at 1.5 hpf. The *mych* expression can be rescued by Pou5f1-VP16 in both, CHX treated and untreated embryos, and therefore the regulatory influence of Pou5f1 should be direct (A-B). The expression of *mycl1b* can also be rescued by Pou5f1-VP16 overexpression in CHX untreated MZ*spg* embryos, but is also strongly upregulated in CHX treated MZ*spg* embryos, even without the injection of *pou5f1-VP16* mRNA (C-D). Thus the experiment cannot proove whether activation of *mycl1b* by Pou5f1 is direct or not. We used *notail* as negative control, because its expression is independent of Pou5f1 function (E), and depends on zygotic gene products.

To determine the spatial extent of *myc* gene regulation by Pou5f1 in the embryo, and to confirm the microarray data, we addressed experimentally whether and to what level Pou5f1 may be able to directly activate *mych* and *mycl1b* expression. We microinjected mRNA encoding a fusion protein of Pou5f1 with the strong transcriptional activator domain VP16 [49] into MZ*spg* embryos, and inhibited translation of zygotically expressed mRNAs by CHX. Therefore, the effect of Pou5f1-VP16 on *mych* and *mycl1b* should be direct, and not mediated by indirect effects of Pou5f1 targets on *mych* and *mycl1b*. The amount of *pou5f1*-*VP16* mRNA injected was chosen such that the embryos showed a partial rescue of the morphological MZ*spg* phenotype as described by Lunde et al. [49] (Figure S4B), which demonstrates the functionality of the used fusion mRNA. To reveal the specificity of the experiment, we used *no tail* as a control (Figure 3E), which has been shown not to be regulated by Pou5f1 [49], and is not affected by *pou5f1*-*VP16* mRNA in our experiment. We analyzed the expression of *mych* and *mycl1b* by WISH at 60% epiboly. Injection of *pou5f1-VP16* fusion mRNA into MZ*spg* embryos induced strong *mych* and *mycl1b* expression (Figure 3A and C, Supplemental Table S1) also in the presence of CHX (Figure 3B and D). However, *mycl1b* RNA levels were not only increased by pou5f1-VP16 overexpression in CHX treated MZ*spg* embryos, but also by the CHX treatment alone in control MZ*spg* embryos (Figure3 D). Given the strong maternal expression (Figs. 2 and S3), CHX treatment may indirectly prevent degradation of maternal *mycl1b* RNA by mechanisms depending on translation of zygotic protein products.

**Figure 4:**
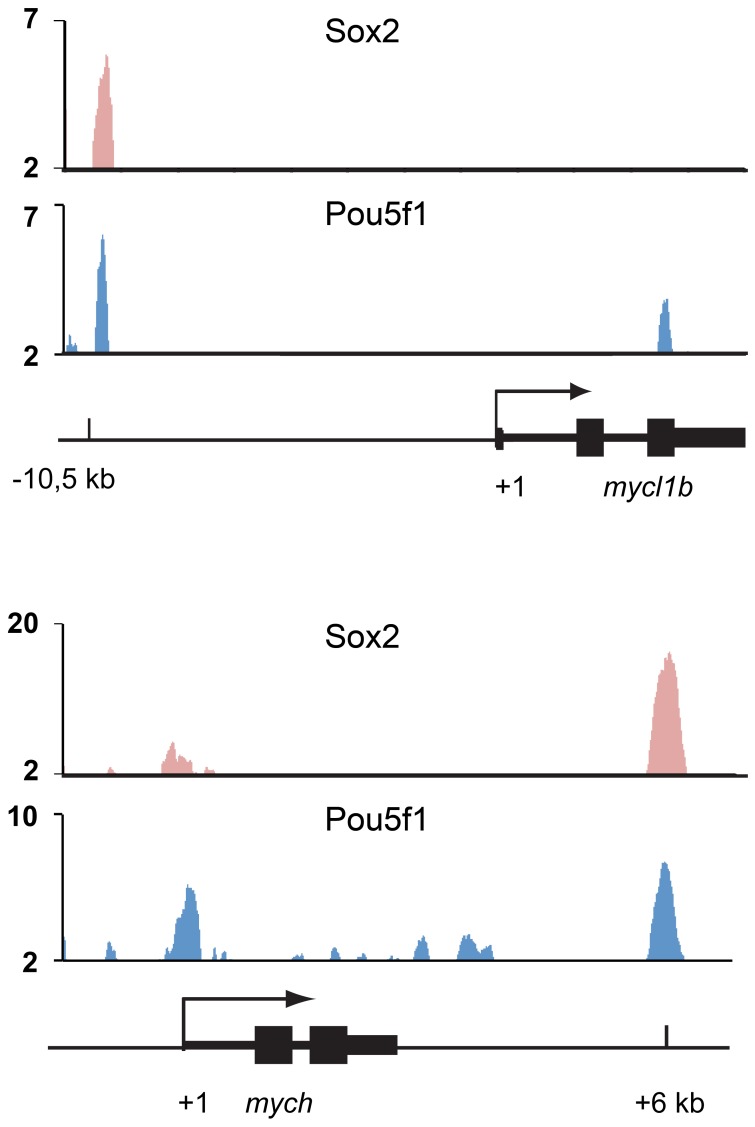
Pou5f1 binds to the promoters and regulatory regions of *mych* and *mycl1b*. ChIP-seq reads for Sox2 (red) and Pou5f1 (blue) [50] mapped to the *mycl1b* (A) and *mych* (B) genetic loci are shown. Colocalisation of Pou5f1 (blue) and Sox2 (red) at the regulatory regions of *mycl1b* and *mych* were detected. Y-axis: ChIP-Seq reads per base, y-axis floor is set to 2.

To investigate whether Pou5f1 may bind to the *mych* and *mycl1b* regulatory regions, we analyzed published genome-scale chromatin immunoprecipitation data (ChIP-seq) for Pou5f1 [50]. Figure 4 shows that at 50% epiboly stage Pou5f1 was detected bound in the proximity of both the *mych* and *mycl1b* genes (+6 kb and -10,5 kb from transcription start sites, respectively). In addition, smaller Pou5f1 binding peaks marked the basal promoter region of *mych*. Three Pou5f1 ChIP-Seq peaks also correlated with Sox2 ChIP-Seq peaks, suggesting that Pou5f1 and Sox2 act together in this regulation. In summary, our data reveal that zygotic transcription of *mych* and *mycl1b* are directly regulated by Pou5f1.

### Pou5f1 mutants show enhanced apoptosis

**Figure 5:**
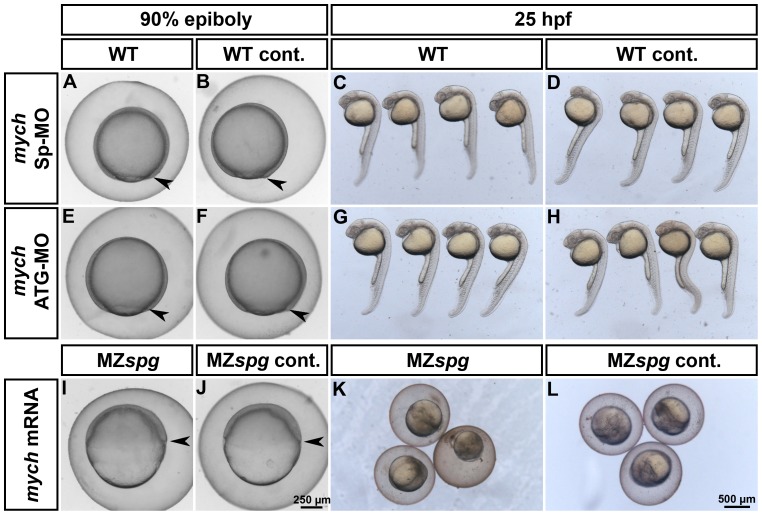
Analysis of the Mych contribution to the morphology of the MZ*spg* mutant phenotype. Knockdown of Mych function in WT by injecting splice-blocking (A-D) or translation-blocking (E-H) morpholinos. Morphological analysis of injected (A,C,E,G) and non-injected (B,D,F,H) embryos at 90%-epiboly and 25 hpf. Morphants show neither a delay in epiboly (A, E) nor obvious morphological defects by 25 hours development (C, G). Rescue of Mych activity in MZ*spg* mutants by injecting *mych* mRNA into 1-cell stage embryos (I-L). Mych alone is not able to rescue the strong defects developed by Pou5f1 deficient embryos during gastrulation (I) and after 25 hpf (K).

The most prominent early phenotype of MZ*spg* embryos is delayed and ultimately arrested progression of epiboly (compare Figure 5B and J) [49], [51], [52]. Pou5f1-dependent regulation of cell adhesion and cell motility has been shown to contribute to the epiboly delay phenotype in MZ*spg* [53], but does not appear to explain the full extent of phenotypic abnormalities in MZ*spg*. Additional causes for the epiboly delay phenotype may include slowdown of cell proliferation or increase in apoptosis. Lachnit et al. [51] demonstrated that until 5 hpf stage there is no reduction in number of deep cells detectable in MZ*spg* mutants compared to wildtype. However, cell death and proliferation have not been analyzed during mid to late gastrula stages in MZ*spg* so far.

We analyzed cell proliferation at 90% epiboly by calculating the mitotic index in MZ*spg* mutants and WT embryos (Figure S5). We fixed embryos and stained chromatin using the Sytox Green fluorescent dye. We recorded animal view confocal stacks of the stained embryos and determined the total number of nuclei as well as the number of metaphase and anaphase nuclei, which characterize cells in mitosis. We calculated the ratio of mitotic nuclei and total nuclei (Figure S5). We found that even at 90% epiboly stage, when the MZ*spg* epiboly phenotype is very pronounced, the mitotic index of the MZ*spg* mutants did not differ significantly from WT embryos (Figure S5A, Supplemental Table S3). Thus, a reduction in cell number caused by decreased proliferation during late gastrulation may be excluded as cause for the MZ*spg* epiboly phenotype.

**Figure 6:**
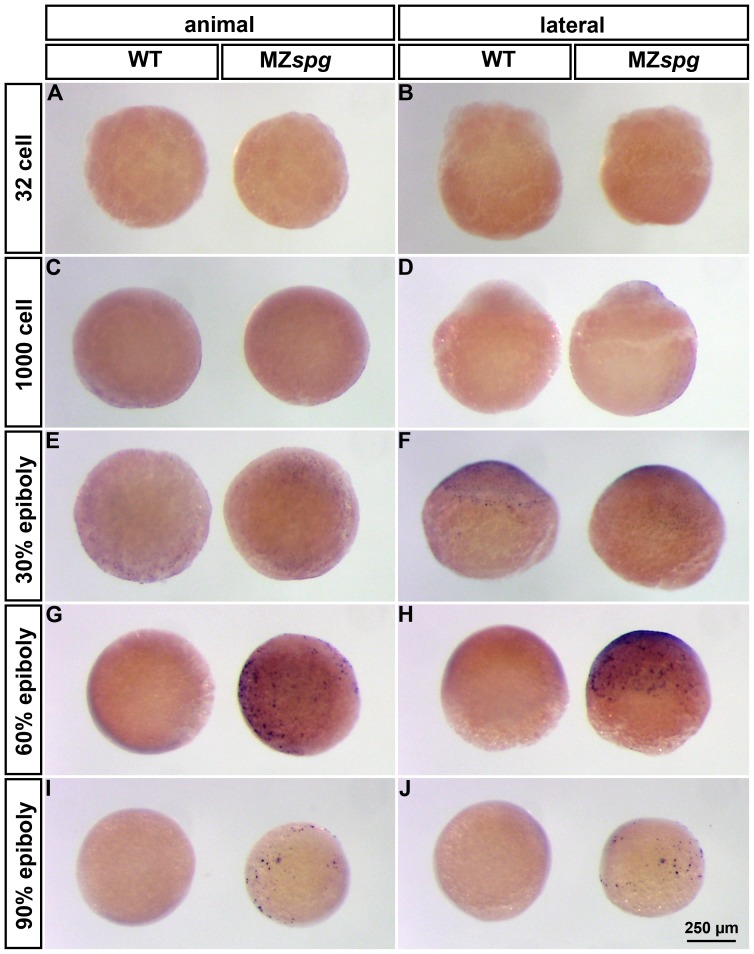
MZ*spg* mutants have enhanced apoptosis during gastrulation. TUNEL staining of WT (left embryo in each panel) and MZ*spg* (right embryo in each panel) embryos at distinct embryonic stages between 32 cells and 90%-epiboly. Embryos are shown in animal (left column) and lateral (right column) view. Pou5f1 deficient embryos show enhanced apoptosis, starting at 60%-epiboly, compared to WT (G-J).

**Figure 7:**
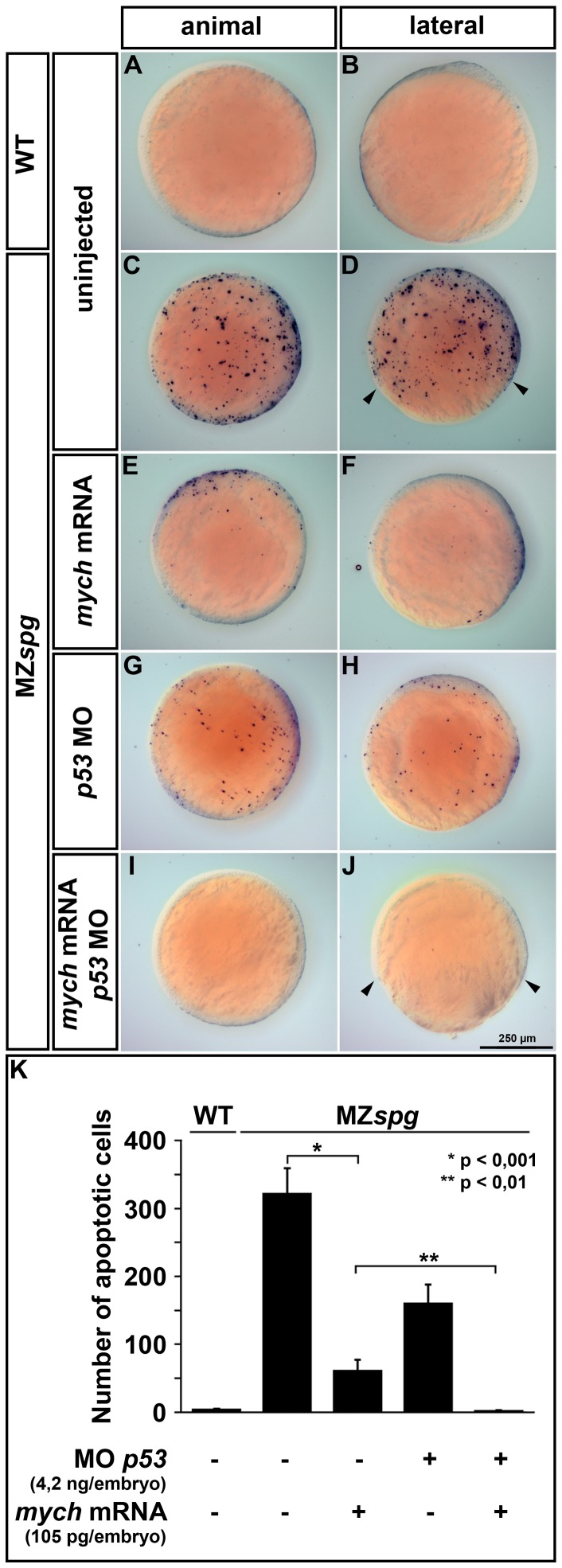
Mych overexpression and p53 knockdown suppress cell death in MZ*spg* gastrulae. TUNEL staining to detect apoptosis at bud stage (A-J) and subsequent computational image analysis (K) for quantification of the number of apoptotic cells. The images show maximum intensity projections of z-stacks taken from single embryos with dorsal to the right. WT embryos display almost no apoptosis, whereas MZ*spg* mutants show an increase in cell death throughout the embryo (A-D). This mutant apoptosis phenotype was partially repressed either by *mych* overexpression (E,F) or p53 morpholino knockdown (G,H). The co-injection of *mych* mRNA and p53-morpholino could completely suppress cell death in MZ*spg* mutants, but did not rescue the delay in epiboly movement (I-J; arrowheads). The quantification of cell death (K) revealed that the number of apoptotic cells is decreased by a factor of six in MZ*spg* embryos after *mych* mRNA injection. By combined knockdown of p53 and Mych overexpression, apoptosis in MZ*spg* embryos was reduced to WT levels.

Controlled cell death, and specifically apoptosis, contributes to embryonic development across the animal kingdom [54]. Cole and Ross [55] described the temporal and spatial distribution of apoptotic cells during normal zebrafish development. The earliest apoptotic cells in wildtype development were reported around 12 hpf in the dorsal midline and the segmental plate. To investigate whether MZspg embryos have enhanced apoptosis before this time stage, we analyzed apoptosis in MZ*spg* mutant and WT embryos using TUNEL assay staining from 32-cell stage up to bud stage (Figs. 6 and 7). We could not detect any apoptotic cells in both MZ*spg* and WT embryos until early gastrula stage (Figure 6A-F). Later in development, starting at 60% epiboly, we found enhanced apoptosis in MZ*spg* mutants, whereas WT embryos showed no TUNEL staining (Figure 6G-J and Figure 7A-D).

### Mych controls apoptosis in zebrafish

To investigate a potential contribution of loss of Mych to the MZ*spg* phenotype we analyzed the morphology after *mych* overexpression in MZ*spg* mutants and Mych knockdown in WT embryos at 90% epiboly and 25 hpf (Figure 5). The knockdown of Mych activity using a splice blocking (Sp-MO) morpholino, which blocks splicing of the second intron of the zygotic *mych* pre-mRNA, or a translation blocking (ATG-MO) morpholino, which blocks the translation of both maternal and zygotic *mych* mRNAs, did not cause a delay of epiboly movement (Figure 5A-B and E-F). To demonstrate the functionality of our *mych* ATG-MO, we knocked down GFP in embryos injected with *gfp* mRNA fused to the *mych* ATG-MO binding site (Figure S6A-H). The effectiveness of our *mych* Sp-MO was shown by *mych* RT-PCR after injecting different concentrations of the splice blocking morpholino (Figure S6I). Also, broad overexpression of *mych* in MZ*spg* mutants by mRNA injection could not rescue the MZ*spg* phenotype (Figure 5I-J). One-day-old manipulated embryos developed morphologically similar to WT or MZ*spg* control embryos, respectively (Figure 5 C-D, G-H and K-L; Supplemental Table S2). A more detailed analysis of the late phenotypes of Mych morphants was reported by Hong et al. [45].

Next, we analyzed whether Mych activity may contribute to control of apoptosis during gastrula stages. Following *mych* knockdown and analysis by TUNEL assay, we found enhanced apoptosis in Mych morphants at 24 hpf (data not shown), comparable to the results reported by Hong et al. [45]. To analyze whether *mych* overexpression can rescue the apoptosis phenotype, we injected *mych* mRNA into one-cell stage MZ*spg* embryos, and compared the numbers of apoptotic cells at late gastrula stage between injected and non-injected embryos. We found that *mych* mRNA injection led to a reduced number of apoptotic cells in the MZ*spg* mutants at bud stage (Figure 7E-F). In parallel we performed p53 knockdown in MZ*spg* embryos, using a p53 specific morpholino, which has been reported to block apoptosis [56]. MZ*spg* p53 MO injected embryos showed a strong reduction in apoptosis compared to non-injected MZ*spg* controls (Figure 7G-H). The co-injection of *mych* mRNA and p53-MO completely rescued the MZ*spg* apoptosis phenotype (Figure 7I-J). We quantified the numbers of apoptotic cells in these experiments (Figure 7K, Figure S7, Supplemental Table S4, Methods). When MZ*spg* embryos injected with *mych* mRNA were compared to control MZ*spg* embryos, a statistically significant six-fold reduction of apoptotic cells in *mych* overexpressing MZ*spg* was observed. p53 knockdown caused a less pronounced reduction of the apoptotic phenotype in MZ*spg*, and the combined overexpression of *mych* and knockdown of p53 rescued the apoptosis essentially back to low wildtype levels (Figure 7K). While we microinjected well-established amounts of *p53* MO for knockdown, and a relatively large amount of *mych* mRNA for rescue, we cannot completely exclude that incomplete rescue or knockdown may contribute to the apparent partial rescue of apoptosis only. We conclude that Mych and p53 dependent pathways are likely to contribute in parallel to the MZ*spg* apoptotic phenotype.

## Discussion

Myc proteins contribute to the control of cell proliferation by regulating cell cycle progression and apoptosis, and play important roles in cancer [28] as well in establishment of pluripotency [37]. Here we investigated the control of *myc* gene expression by the Pou5f1/Oct4 pluripotency factor in the early zebrafish embryo. We found that early zygotic *mych* expression as well as late gastrula stage *mycl1b* expression both directly depend on Pou5f1 activity. We further showed that Pou5f1 deficient MZ*spg* embryos developed enhanced apoptosis already during early gastrula stages, and that *mych* overexpression in MZ*spg* embryos was able to significantly suppress the apoptosis phenotype. Combined knockdown of p53 and overexpression of Mych completely rescued the MZ*spg* apoptosis phenotype. Both results together reveal that Mych has anti-apoptotic activity in the early zebrafish embryo, and that p53-dependent and Myc pathways act in parallel to control apoptosis at these stages.

### Zebrafish *myc* genes are broadly expressed during the first hours of development

Our and others previous studies have shown that zebrafish L-*myc* and c-*myc* homologous genes are maternally expressed and mRNAs deposited in the egg, which remain stable until blastula stages [42], [44], [45], whereas *mycn* and *mych* expression are first detectable after mid-blastula transition [44]. Zebrafish *mycl1a, mycl1b*, and *mych* genes are broadly expressed in blastoderm cells after MBT. Broad *myc* gene expression during early development was also shown in other vertebrate species. The *Xenopus* c-*myc*, L-*myc* and N-*myc* homologous genes are all maternally expressed, but their expression levels decrease during early embryogenesis [57], [58]. xc-*myc* expression is maintained at a low level during blastula and gastrula stages before it increases again during neurulation. During mouse gastrulation c-*myc* and N-*myc* are widely expressed in embryonic and extraembryonic tissues [32]. Thus, the broad *myc* gene expression during blastula and gastrula stages is evolutionary conserved throughout the vertebrate subphylum. However, not much is known about potentially conserved *myc* gene functions during blastula and gastrula stages.

### Pou5f1 activity is required for proper *mych*, *mycl1b* and *mycn* expression

We found that the transcription factor Pou5f1 is required for proper transcription of *mych* after MBT from the 1000-cell stage on. While Pou5f1 appears to directly activate the broad early zygotic expression of *mych* after MBT, Pou5f1 activity is not required for the initiation of the later *mych* expression domain in the involuted axial mesoderm on the dorsal side of the embryo. Pou5f1 protein is also involved in proper maternal regulation of *mycl1b*, as enhanced levels of *mycl1b* were detected at pre-MBT and blastula stages in MZ*spg*. Further, Pou5f1 is required for proper maintenance of *mycl1b* expression level during mid- to late gastrula stages, when *mycl1b* drops significantly below wildtype levels in MZ*spg* embryos. In addition, Pou5f1 is required for proper early post-MBT activation of *mycn* expression, which is delayed in MZ*spg* mutants.

The regulation of c-*myc* expression has been intensively studied in several systems. A number of regulatory *cis*-elements have been identified in mammals and it was shown that many transcription factors can bind to the c-*myc* regulatory elements *in vivo* [59]. Chen et al. [60] analyzed the core transcriptional network of embryonic stem cells using chromatin immunoprecipitation coupled with ultra-high-throughput DNA sequencing (ChIP-seq) to map the locations of key pluripotency transcription factors like Oct4, Sox2, Nanog, c-Myc and n-Myc. They could show that Oct4 and Sox2 directly bind to the n-*myc* promoter region, but do not interact with c-*myc*. This reported regulation of *myc* family members is consistent with our results in early zebrafish embryogenesis, where *mych* and *mycl1b* genes are directly induced by Pou5f1 and potentially also bound by Sox2. Thus, regulation of *myc* gene expression by Pou5f1 and Sox2 during early embryogenesis, respectively in ES cells, may be an evolutionary conserved feature in vertebrate development.

### Maternal and zygotic *pou5f1* mutants show normal proliferation, but enhanced apoptosis at gastrulation stages

When analyzing cell division rates and cell survival at blastula to gastrula stages, we found no differences in the mitotic rates between MZ*spg* and WT embryos, while apoptosis rates were markedly increased in MZ*spg* embryos starting from 60% epiboly. The relationship of Oct4 to apoptosis in mammalian ES cells is not well understood. Oct4 is a central factor in the ES cell transcriptional network, controlling many parameters of ES cell biology [38], [40]. This role is compatible with both promoting proliferation and inhibiting apoptosis, as ES cells are immortal cell population with fast self-replication rates. Oct4 deprivation results in global expression changes, cessation of rapid cell proliferation and finally differentiation of ES cells to trophectoderm. However, Oct4 has also been linked to survival and anti-apoptotic pathways in ES cells by several different potential mechanisms: by a STAT3/Survivin route [61], by Trp53 regulation [62], or by a miR-125b pathway [63]. Next to its role in ES cells, Oct4 has also been suggested to promote survival of cancer stem cells by inhibiting apoptosis [63], [64].

In zebrafish, like in mammals, Pou5f1 controls expression of a large network of developmentally important signaling molecules and transcription factors [47], [50]. The majority of these factors is expressed in embryonic tissue-specific patterns, starting already from mid-gastrulation stages [47], [49], [50], [65]. Therefore, it is possible, that the absence of some of these factors may not be compatible with cell survival within a specific tissue, which may lead to the activation of safeguarding apoptotic mechanism. However, we did not detect any obvious tissue-specific pattern of apoptosis in MZ*spg* mutants, which suggests that Pou5f1 may be required throughout the whole embryo to prevent the activation of apoptotic cascades.

### Mechanisms of anti-apoptotic action of Pou5f1: separate Myc and p53 branches

The expression of multiple *myc* genes, *mych*, *mycl1b*, and to a lesser degree *mycn*, is reduced in MZ*spg* embryos. Since *myc* genes are known anti-apoptotic factors acting through different routes including p19^ARF^, BIM and BCL2 (reviewed in [66]) and MDM2 [67], they may convey the anti-apoptotic action of Pou5f1. Enhanced early apoptosis in MZ*spg* may be caused by reduced Myc activity, where individual *myc* genes may act partially redundantly. Indeed, we found that *mych* mRNA overexpression is able to rescue most of the ectopic apoptosis in MZ*spg* embryos. However, the knockdown of *mych* in wildtype embryos did not induce apoptosis during gastrulation stages (10 hpf stage; data not shown), strengthening the notion that *mych*, *mycl1b* and *mycn* gene activities, which are all broadly expressed in the blastula and gastrula embryo [44], [45], may be required redundantly downstream of Pou5f1 to prevent early activation of the apoptotic programs.

p53, together with co-factors and depending on the type of stress a cell is subjected to, is a universal activator of apoptosis [68], acting in response to various stimuli. In many cell systems, *myc* genes contribute to control of apoptosis by regulating p53, e.g. in a p19^ARF^ dependent manner [66]. We determined whether Pou5f1 may regulate levels of *tp53* mRNA by reanalyzing published time-series microarray data for MZ*spg* and WT embryos [47], and found no significant differences in *tp53* mRNA levels from zygote to end of gastrulation. Thus, Pou5f1 does not regulate p53 expression, and an involvement of p53 may be through indirect pathways controlling p53 activity. If all apoptosis in MZ*spg* would be p53-dependent, apoptosis should be completely abolished by knockdown of p53 through injection of p53-morpholino. However, in p53*-*MO injected MZ*spg* embryos, as in *mych* mRNA injected embryos, apoptosis was found to be only partially suppressed. Complete suppression of apoptosis in MZ*spg* embryos was achieved only by simultaneous increase of Mych activity and suppression of p53. This suggests that *myc* genes, specifically *mych*, in the early zebrafish embryo are able to suppress apoptosis through a p53-independent pathway. A similar p53-independent mechanism may potentially be involved in the suppression of apoptosis in the neural plate by Mych at later developmental stages [45]. Our study suggests that the zebrafish embryo may be a suitable model system to dissect p53-dependent and independent anti-apoptotic activities of Myc proteins during embryonic development. However, the coexpression of several *myc* genes throughout the early embryonic stages will likely require combined inactivation of each of these genes, which so far has hindered progress towards analysis of molecular mechanisms.

## Materials and Methods

### Ethics statement

This study was performed with the approval of the State of Baden-Württemberg Regierungspraesidium Freiburg Animal Protection Authorities in accordance with the German Animal Protection Act under permission number 35-9185.81/G-12/40.

### Fish and embryo care

We used WT embryos of AB x TÜB strain crosses (http://www.ZFIN.org) and MZ*spg* embryos carrying the *m793* allele of the *spg* mutation [69](ZFIN ID: ZDBGENE-980526-485, ZDB-GENO-081023-1). Fish were raised, maintained and crossed under standard conditions as described [70]. Embryos were incubated or raised in egg water or in 0.3× Danieau's solution at 28.5°C. Developmental age is reported as hours post fertilization (hpf) when incubated at 28.5°C. Developmental stages of MZ*spg* embryos were indirectly determined by observation of WT embryos born at the same time and incubated under identical conditions.

### Morpholinos

Morpholino oligos *mych*-Sp-MO: 5′-GTAGCAAAAGACTCACCAGAATCGC-3′, *mych*-ATG-MO: 5′-GCAGCATCTTGACGGAACCTTTTTC-3′, and standard control morpholino SCMO: 5′-CCTCTTACCTCAGTTACAATTTATA-3′ were ordered from Gene Tools (Philomath, USA). The *mych*-ATG-MO blocks the translation of the *mych* mRNA into protein by binding to the translation start site. The *mych*-Sp-MO prevents splicing of the second intron resulting in a non-functional truncated protein missing the DNA binding domain. To test the specificity of *mych*-ATG-MO, the sequence -4 to +21 bp from the *mych* translation start site ATG was cloned upstream of GFP ORF in the CS2+ vector, to obtain the *mych*-GFP construct. 50 pg/embryo of in *vitro* transcribed *mych*-GFP mRNA was injected into one-cell stage embryos together with 1.4 ng, 4.1 ng or 8.6 ng per embryo of the *mych*-ATG-MO or without Morpholino (Figure S6). The efficiency of *mych*-Sp-MO was tested by injecting 1.4 ng, 4.1 ng or 8.6 ng into single cell WT embryos and subsequent RT-PCR analysis at 60% epiboly stage. The ratio of correctly spliced *mych* mRNA (162 bp) and mRNA containing the second intron (396 bp) shows that the injection of 4.1 ng Morpholino is sufficient to nearly completely inhibit splicing (Figure S6). To address the contribution of *mych* to epiboly delay phenotype in MZ*spg* mutant embryos we injected 4.6 ng of *mych*-Sp-MO and 1.4 ng *mych*-ATG-MO into 1-cell stage embryos respectively. As control we injected the same amount of SCMO.

### Cycloheximide experiment

MZ*spg* embryos were injected with 10 pg *pou5f1-VP16* mRNA at the 1-cell stage or left non-injected as controls. Embryos were treated with 15 mg/ml of cycloheximide (CHX, Calbiochem) dissolved in egg water. CHX was added at 1.5 hpf to allow for translation of injected mRNAs, but to block translation of the earliest zygotic transcripts. In the presence of CHX, direct Pou5f1 targets are transcribed after MBT, but these mRNAs are not translated, avoiding indirect downstream regulatory effects. Loss of *ntl* expression in CHX embryos was used as control for efficient inhibition of translation [71].

### Plasmids used in this study

The zebrafish expression construct CS2+ Pou5f1-VP16 has been described [49]. The following *myc* gene EST clones were ordered from RZPD (Deutsches Ressourcenzentrum für Genomforschung GmbH): IMAGp998E208991Q1 (*mych*), IRBOp991F125D2 (*myca*), IRBOp991G0414D2 (*mycb*), IRBOp991E0238D2 (*mycl1a*), IRAKp961I20283Q2 (*mycl1b*).

### Whole-mount *in situ* hybridization and *in situ* detection of apoptosis

Whole-mount *in situ* hybridization was performed as described [72].

The ApopTag Peroxidase In Situ Apoptosis Detection Kit (Chemicon/Merck Millipore) was used to detect apoptotic cells in early embryonic stages. To investigate whether MZ*spg* embryos develop enhanced apoptosis, MZ*spg* and WT embryos were fixed in 4% PFA at several developmental stages between 32-cell and bud stage. The fixed embryos were incubated in 100% methanol to make them permeable for the TUNEL staining. To analyze the influence of Mych and p53 on this phenotype, 105 pg *mych* mRNA and/or 4.2 ng *p53*-MO were injected at one-cell stage into MZ*spg* embryos. Non-injected WT and MZ*spg* embryos were used as controls. Embryos were fixed at 10 hpf. To quantify apoptosis image z-stacks of 5-14 stained embryos were taken for each experiment using transmitted light microscopy. Thereafter, maximum intensity projections of z-stacks were calculated for each embryo using ImageJ (Rasband, W.S., ImageJ, U.S. National Institutes of Health, Bethesda, Maryland, USA, http://imagj.nih.gov/ij/, 1997–2012) and apoptotic nuclei were automatically detected using the following set of parameters in Volocity Image Analysis Software (PerkinElmer): (1) Find Objects by Intensity; (2) exclude objects smaller than 10 μm^2^; (3) separate touching objects greater than 50 μm^2^ (Figure S7).

### Cell proliferation analysis

To determine a potential role of Mych in proliferation, 105 pg *mych* mRNA and/or 4.2 ng *p53*-MO were injected into one-cell stage MZ*spg* embryos. Non-injected WT and MZ*spg* embryos were used as controls. Embryos were fixed in 4% PFA at 10 hpf. The embryos were incubated in 100% methanol to make them permeable for the Sytox-Green DNA stain. Embryos were stained in 2 μM Sytox-Green (Invitrogen) at room temperature (20 to 25°C) for 2 hours. Mitotic nuclei (prometa-, meta- and anaphase nuclei) were manually detected by their characteristic chromosome arrangement and dense DNA structure, resulting in higher Sytox-Green intensity. The mitotic index is the proportion of mitotic nuclei to the total number of nuclei. For comparisons WT mitotic index was normalized to 1.

### Microinjections

mRNAs were synthesized using the mMessage mMachine kit (Invitrogen) according to manufacturer's instructions. mRNA or morpholino were injected into the yolk of freshly fertilized zygotes (younger than 15 minutes) mounted on 1% (w/v) agarose ramps, using microinjection pipettes connected to an air pressure driven microinjector. A volume of 0.5–1 nl, containing mRNA or morpholino and 0.5% (v/v) phenol red in water, was injected into each zygote.

### Quantitative RT-PCR

60–100 embryos per sample were snap-frozen in liquid nitrogen, and total RNA was isolated using the RNA Easy kit (Qiagen). cDNA was synthesized using Superscript III kit (Invitrogen). cDNA was amplified using gene-specific primers and ABsolute SYBR Green Fluorescein (ABgene, Thermo Scientific) according to manufacturer's instructions on an Bio-Rad i-Cycler. Results were calculated using the ddCT method and zebrafish *ef1α* as a normalization control. Primers used: *mych* forward 5′-CCAGGCACTGGAGAGCGAAGAC-3′, reverse 5′-AGAGCGTGTCAGGGTGCTGGAG-3′; *mycl1b* forward 5′-GGTCAGAATCTCGCACCGACTC-3′, reverse 5′-ATGTGGAAACGCTTCATGCAGG-3′; and *ef1α* forward 5′-CCTGGGAGTGAAACGCTGATC-3′, reverse 5′-CCGATCTTCTTGATGTATGCGCTG-3′. Calculations are based on Pfaffl [73].

### Statistical analysis

Error bars in Figures show Standard Error of the Mean (SEM). Significance was evaluated using Student's T-test (Microsoft Excel).

## Supporting Information

Figure S1 **Phylogenetic analysis of the six zebrafish** ***myc*** **genes.** Phylogenetic tree of *myc* family genes. In zebrafish two paralogous genes each exist for *L-myc, mycl1a and mycl1b*, and *c-myc, myca* and *mycb*. In addition, there is a single copy each for the *mycn* and *mych* genes. The later one is closely related to the *N-myc* and *c-myc* genes, but has no known homologues in other vertebrate species. Trees were built using phylip proml, and 100 datasets for bootstrapping. The alignment was done with clustalw. Sequences used are: *hs-nmyc* NP_005369.2, *mm-nmyc* NP_032735.2, *ha-myc* NP_002458.2, *mm-myc* NP_034979.3, *hs-lmyc* NP_001028253.1, *mm-lmyc* NP_032532.1, *hs-smyc* E10909, *mm-smyc* NP_034980.1, *mm-bmyc* NP_075815.2, *dr-myca* NP_571487.2, *dr-mycb* NP_956466.1, *dr-mych* XP_005166306.1, *dr-nmyc* NP_997779.1, *dr-mycl1a* NP_998102.1, *dr-mycl1b* NP_001038607.1.
(TIF)

Figure S2 **WISH analysis of** ***myc*** **gene expression at 24 hpf.** WISH analysis of *myca*, *mycb*, *mych*, *mycl1a* and *mycl1b* expression in WT (A-J). All embryos are shown in lateral (left column) and dorsal (right column) view. *notail* expression was used as control to evaluate stain background levels in the head, where *notail* is not expressed (K-L). All *myc* genes show a gene specific expression pattern and are mainly expressed in proliferating and neural tissues. The expression of the c-Myc orthologous genes, *myca* and *mycb* (A-D), and of the L-Myc orthologous genes, *mycl1a* and *mycl1b* (G-J), show partially complementary patterns.
(TIF)

Figure S3 ***myc*** **gene expression profiles and transcriptional regulation by Pou5f1 and Sox2.** (A, C, E, G, I, K) Microarray time series data [47] of *myc* gene expression profiles in WT (white squares) and MZ*spg* (black triangles) within the first 8–hours of development. The highest expression value for each gene was normalized to 100. (B, D, F, H, J, L) Microarray analysis of transcriptomes of MZ*spg* embryos injected with mRNA encoding Pou5f1, and developed in presence of CHX from 64-cell stage on. Data are from [47]. Non-injected MZ*spg* control was normalized to 1.
(TIF)

Figure S4 **Morphological analysis of CHX treated and** ***pou5f1-VP16*** **mRNA injected control embryos.** (A) Morphological phenotype of MZ*spg* embryos treated with CHX from 64-cell stage on and developed until WT control embryos reached 60% epiboly. Treated embryos are arrested before sphere stage, but do not degenerate until 60% epiboly equivalent age. (B) The injection of 10 pg *pou5f1-VP16* mRNA into 1-cell MZ*spg* embryos is sufficient to rescue the MZ*spg* phenotype, but it also may ventralize the embryo as Pou5f1 overexpression in WT would do [74]. The experiment demonstrates that *pou5f1-VP16* was injected in our experiments at concentrations that could be considered physiological for embryonic development.
(TIF)

Figure S5 **Analysis of the mitotic index at 90%-epiboly.** Quantification of the proportion of cells undergoing cell division in WT, MZ*spg* and MZ*spg* injected with *mych* mRNA and/or p53 morpholinos by calculating the mitotic index (ratio between the total number of nuclei and nuclei undergoing cell division). (A). The calculated mitotic indices are not significantly different between the different genotypes and experimental conditions. Mitotic index of WT embryos was set to 1. Confocal microscopy Z-stacks were taken from the animal region of 90%-epiboly stage embryos, whose nuclei are stained by Sytox fluorescent DNA dye (B). Chromatin is highly condensed during meta- and anaphase of the cell division, which leads to an increase in Sytox stain intensity (B; arrows).
(TIF)

Figure S6 **Testing of** ***mych*** **morpholino functionality.** (A-H) The functionality of the *mych* translation-blocking morpholino (ATG-MO) was tested by injecting a fusion mRNA, where the MO target sequence was fused to the *gfp* ORF at the start ATG, together with different concentrations of the ATG-MO into one-cell stage embryos. The GFP signal was analyzed using fluorescence microscopy (left panel) and the normal morphology of the embryos after morpholino injection was documented using transmitted light microscopy (right panel). The translation of *gfp* was completely blocked by injecting as little as 1.4 ng of the ATG-MO (C). For the splice-blocking morpholino (Sp-MO) the functionality was tested by RT-PCR using a pair of primers overlapping the second intron (I), whose splicing sites are targeted by the *mych*-Sp-MO. In WT the 162 bp fragment reflects the proper splicing of the pre-mRNA, whereas after the injection of 4.1 ng or more of *mych*-Sp-MO the detected fragment contains the intron and its size increased to 396 bp (I).
(TIF)

Figure S7 **Quantification of apoptosis in WT and MZ** ***spg*** **embryos at bud stage.** Detection of apoptotic cells by TUNEL staining (A) and subsequent computational image analysis (B). The images show a lateral maximum intensity projection of a z-stack taken from a single embryo. (B) The same z-stack after automatic object recognition using Volocity software (Perkin-Elmer), where most of the apoptotic cells are marked in red (B).
(TIF)

Table S1 ***mych*** **and potentially also** ***mycl1b*** **are directly regulated by Pou5f1.** (Referring to: Figure 3)
(PDF)

Table S2 **Analysis of the Mych contribution to the morphology of the MZ** ***spg*** **mutant phenotype.** (Referring to: Figure 5)
(PDF)

Table S3 **Analysis of the mitotic index at 90%-epiboly by Sytox nuclear stain.** (Referring to: Figure S5)
(PDF)

Table S4 **Mych overexpression and p53 knockdown suppress cell death in MZ** ***spg*** **gastrulae.** (Referring to: Figure 7)
(PDF)
