# Supplementary figures and images for: Pou5f1/Oct4 Promotes Cell Survival via Direct Activation of mych Expression during Zebrafish Gastrulation
Source: PLoS One. 2014 Mar 18;9(3):e92356. doi: 10.1371/journal.pone.0092356 (PMC3958507; doi:10.1371/journal.pone.0092356)

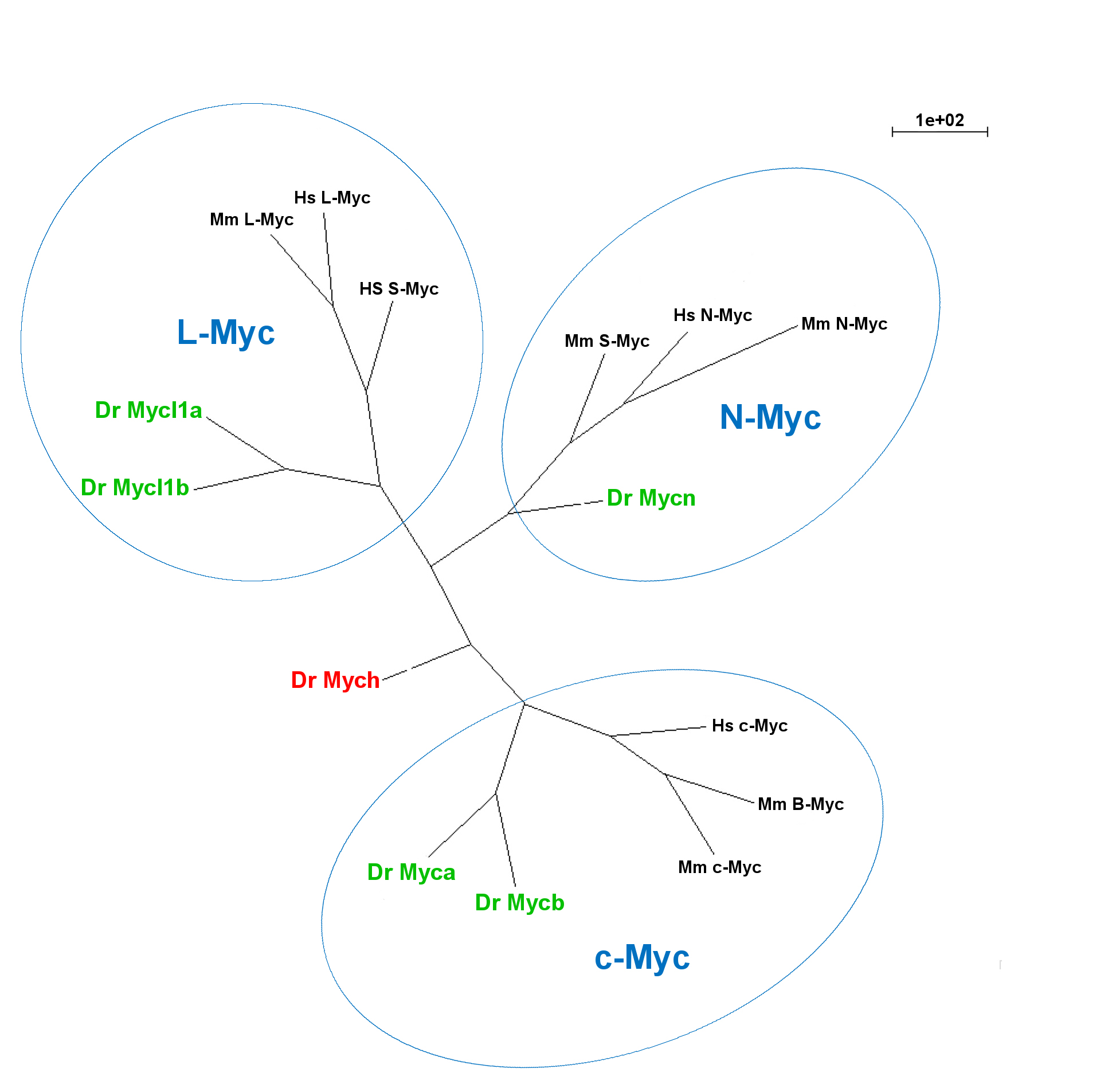

Supplement: Figure S1 — Phylogenetic analysis of the six zebrafish myc genes. Phylogenetic tree of myc family genes. In zebrafish two paralogous genes each exist for L-myc, mycl1a and mycl1b, and c-myc, myca and mycb. In addition, there is a single copy each for the mycn and mych genes. The later one is closely related to the N-myc and c-myc genes, but has no known homologues in other vertebrate species. Trees were built using phylip proml, and 100 datasets for bootstrapping. The alignment was done with clustalw. Sequences used are: hs-nmyc NP_005369.2, mm-nmyc NP_032735.2, ha-myc NP_002458.2, mm-myc NP_034979.3, hs-lmyc NP_001028253.1, mm-lmyc NP_032532.1, hs-smyc E10909, mm-smyc NP_034980.1, mm-bmyc NP_075815.2, dr-myca NP_571487.2, dr-mycb NP_956466.1, dr-mych XP_005166306.1, dr-nmyc NP_997779.1, dr-mycl1a NP_998102.1, dr-mycl1b NP_001038607.1. (TIF) [file pone.0092356.s001.tif]

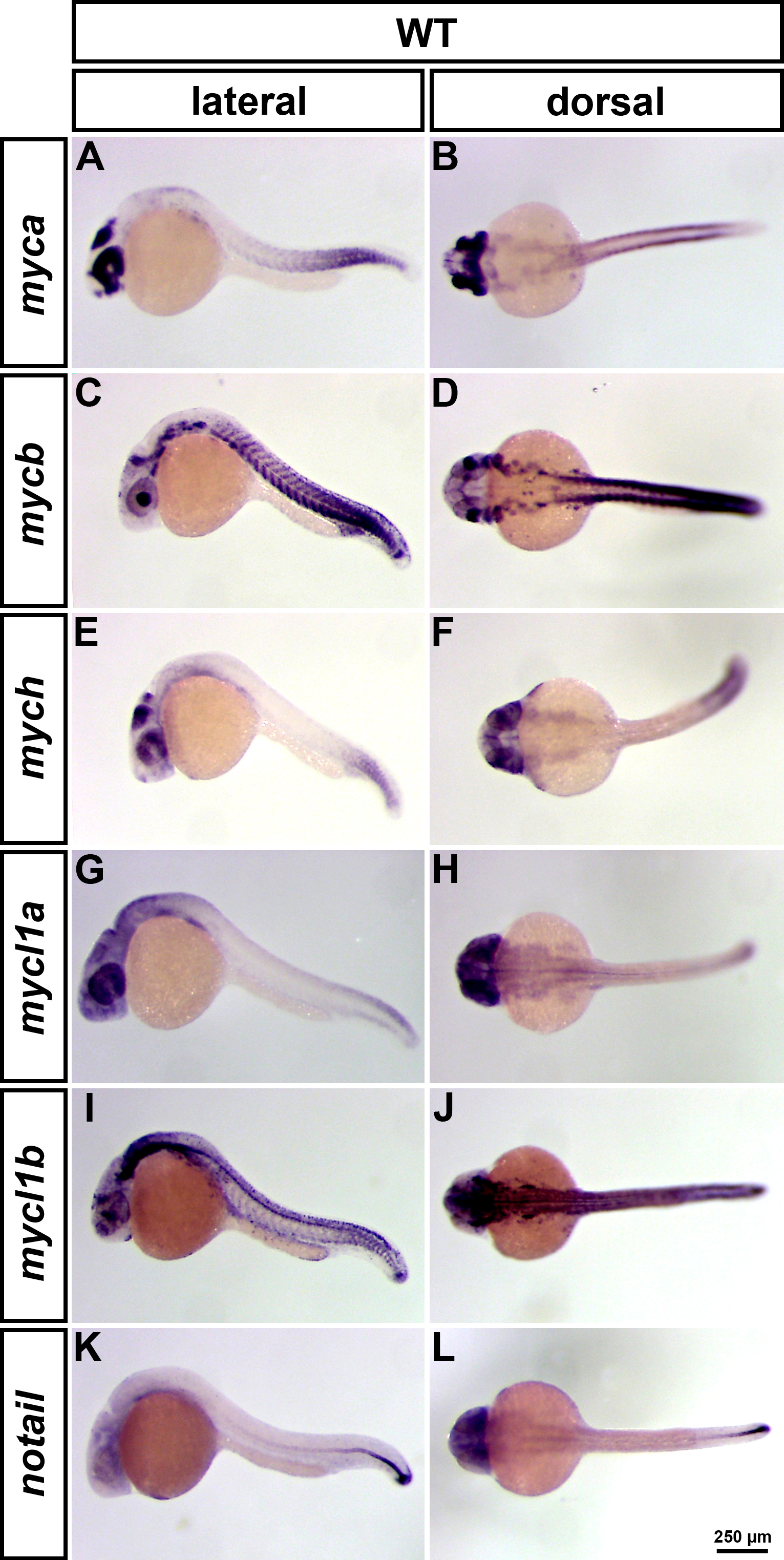

Supplement: Figure S2 — WISH analysis of myc gene expression at 24 hpf. WISH analysis of myca, mycb, mych, mycl1a and mycl1b expression in WT (A-J). All embryos are shown in lateral (left column) and dorsal (right column) view. notail expression was used as control to evaluate stain background levels in the head, where notail is not expressed (K-L). All myc genes show a gene specific expression pattern and are mainly expressed in proliferating and neural tissues. The expression of the c-Myc orthologous genes, myca and mycb (A-D), and of the L-Myc orthologous genes, mycl1a and mycl1b (G-J), show partially complementary patterns. (TIF) [file pone.0092356.s002.tif]

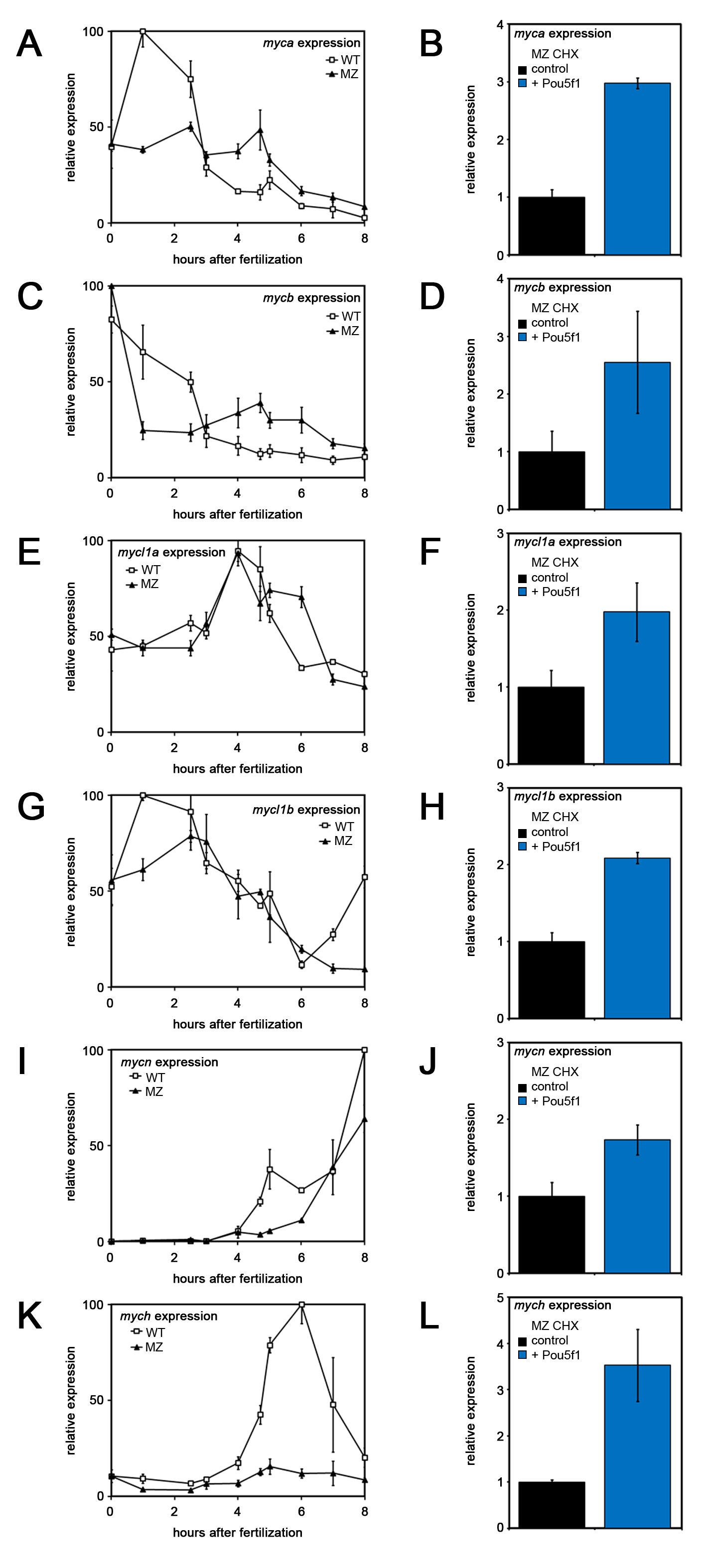

Supplement: Figure S3 — myc gene expression profiles and transcriptional regulation by Pou5f1 and Sox2. (A, C, E, G, I, K) Microarray time series data [47] of myc gene expression profiles in WT (white squares) and MZspg (black triangles) within the first 8–hours of development. The highest expression value for each gene was normalized to 100. (B, D, F, H, J, L) Microarray analysis of transcriptomes of MZspg embryos injected with mRNA encoding Pou5f1, and developed in presence of CHX from 64-cell stage on. Data are from [47]. Non-injected MZspg control was normalized to 1. (TIF) [file pone.0092356.s003.tif]

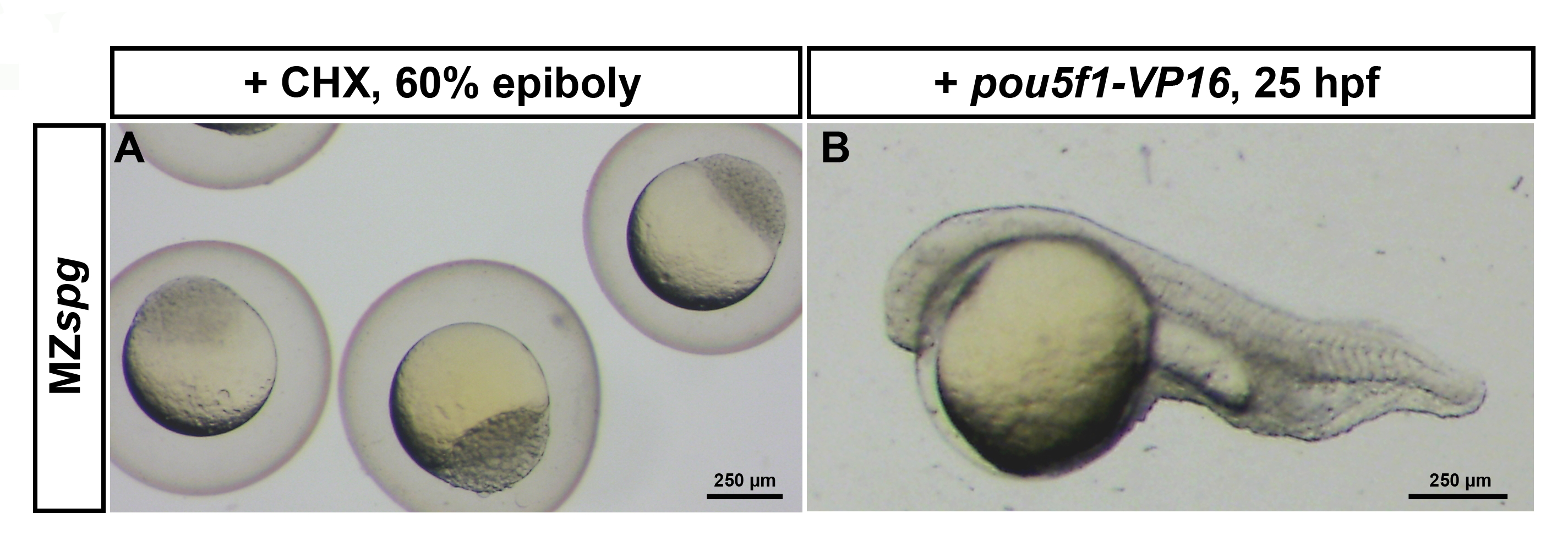

Supplement: Figure S4 — Morphological analysis of CHX treated and pou5f1-VP16 mRNA injected control embryos. (A) Morphological phenotype of MZspg embryos treated with CHX from 64-cell stage on and developed until WT control embryos reached 60% epiboly. Treated embryos are arrested before sphere stage, but do not degenerate until 60% epiboly equivalent age. (B) The injection of 10 pg pou5f1-VP16 mRNA into 1-cell MZspg embryos is sufficient to rescue the MZspg phenotype, but it also may ventralize the embryo as Pou5f1 overexpression in WT would do [74]. The experiment demonstrates that pou5f1-VP16 was injected in our experiments at concentrations that could be considered physiological for embryonic development. (TIF) [file pone.0092356.s004.tif]

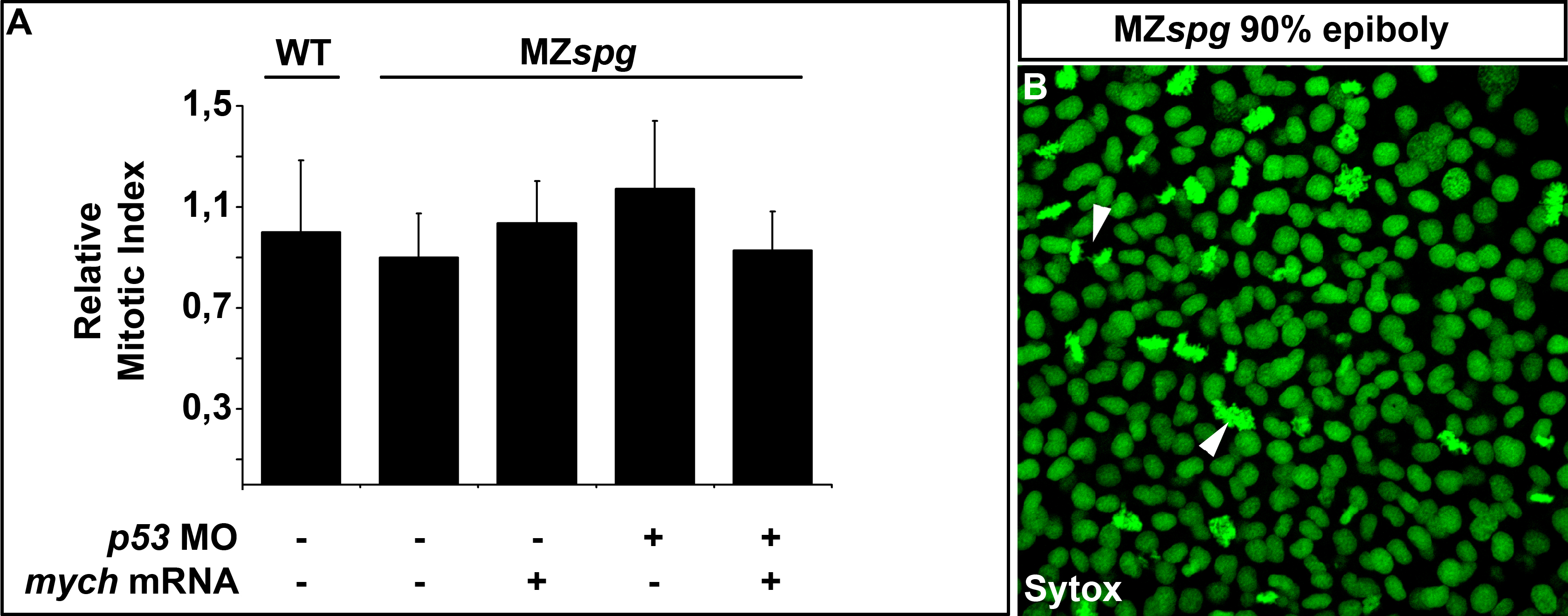

Supplement: Figure S5 — Analysis of the mitotic index at 90%-epiboly. Quantification of the proportion of cells undergoing cell division in WT, MZspg and MZspg injected with mych mRNA and/or p53 morpholinos by calculating the mitotic index (ratio between the total number of nuclei and nuclei undergoing cell division). (A). The calculated mitotic indices are not significantly different between the different genotypes and experimental conditions. Mitotic index of WT embryos was set to 1. Confocal microscopy Z-stacks were taken from the animal region of 90%-epiboly stage embryos, whose nuclei are stained by Sytox fluorescent DNA dye (B). Chromatin is highly condensed during meta- and anaphase of the cell division, which leads to an increase in Sytox stain intensity (B; arrows). (TIF) [file pone.0092356.s005.tif]

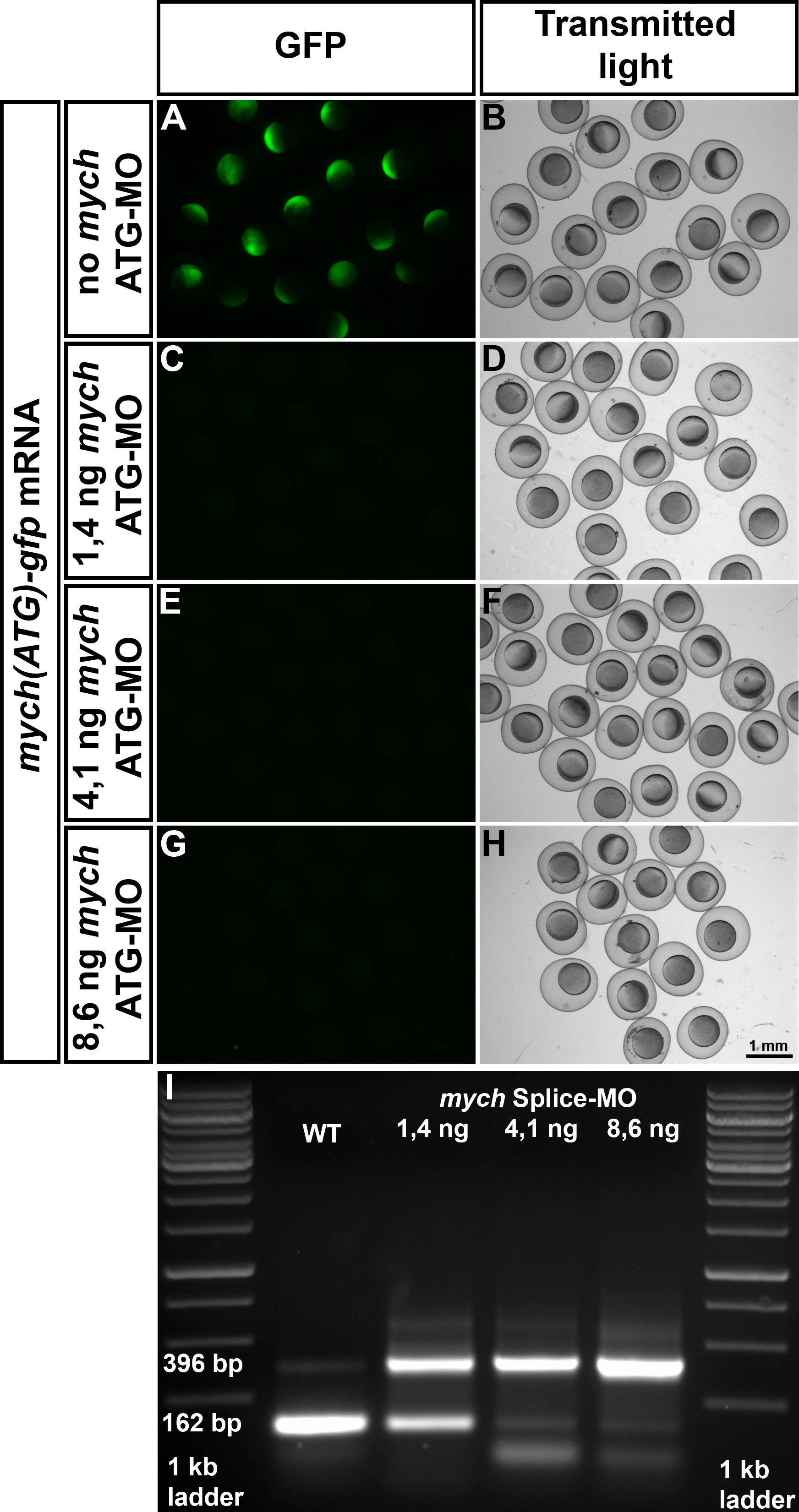

Supplement: Figure S6 — Testing of mych morpholino functionality. (A-H) The functionality of the mych translation-blocking morpholino (ATG-MO) was tested by injecting a fusion mRNA, where the MO target sequence was fused to the gfp ORF at the start ATG, together with different concentrations of the ATG-MO into one-cell stage embryos. The GFP signal was analyzed using fluorescence microscopy (left panel) and the normal morphology of the embryos after morpholino injection was documented using transmitted light microscopy (right panel). The translation of gfp was completely blocked by injecting as little as 1.4 ng of the ATG-MO (C). For the splice-blocking morpholino (Sp-MO) the functionality was tested by RT-PCR using a pair of primers overlapping the second intron (I), whose splicing sites are targeted by the mych-Sp-MO. In WT the 162 bp fragment reflects the proper splicing of the pre-mRNA, whereas after the injection of 4.1 ng or more of mych-Sp-MO the detected fragment contains the intron and its size increased to 396 bp (I). (TIF) [file pone.0092356.s006.tif]

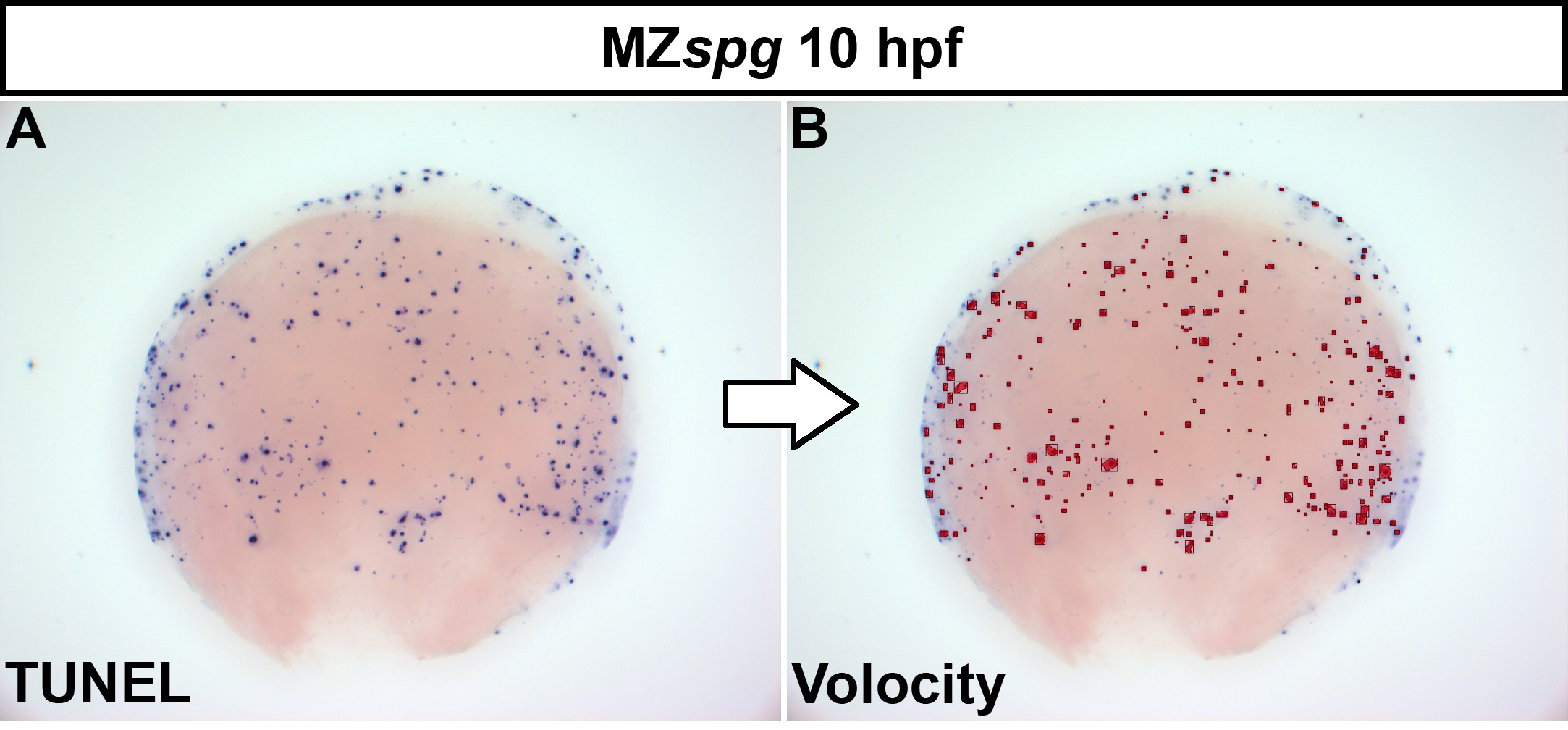

Supplement: Figure S7 — Quantification of apoptosis in WT and MZ spg embryos at bud stage. Detection of apoptotic cells by TUNEL staining (A) and subsequent computational image analysis (B). The images show a lateral maximum intensity projection of a z-stack taken from a single embryo. (B) The same z-stack after automatic object recognition using Volocity software (Perkin-Elmer), where most of the apoptotic cells are marked in red (B). (TIF) [file pone.0092356.s007.tif]
