# Supplementary material for: Pou5f1/Oct4 Promotes Cell Survival via Direct Activation of mych Expression during Zebrafish Gastrulation
Source: PLoS One. 2014 Mar 18;9(3):e92356. doi: 10.1371/journal.pone.0092356 (PMC3958507; doi:10.1371/journal.pone.0092356)
Supplement: Table S1 — mych and potentially also mycl1b are directly regulated by Pou5f1. (Referring to: Figure 3) (PDF) [file pone.0092356.s008.pdf]

Supplemental Table S1

Referring to: Figure 3
mych and potentially also *mycl1b* are directly regulated by Pou5f1

Analysis of *mycl1b* and *mych* expression by WISH											
		WT		MZ*spg*							
10 pg	***pou5f1-VP16*** **mRNA**	**-**		**-**		**+**		**-**		**+**	
15 µg/ml	**CHX**	**-**		**-**		**-**		**+**		**+**	
	**Expression:**	***mych***	***mycl1b***	***mych***	***mycl1b***	***mych***	***mycl1b***	***mych***	***mycl1b***	***mych***	***mycl1b***
**Number of Embryos**	strong expression	20	24	-	-	16	15	-	11	15	15
	weak expression	-	-	16	17	2	5	-	-	-	-
	very weak or no expression	-	-	-	-	-	-	11	-	5	-
	embryos analyzed	20	24	16	17	18	20	11	11	20	15
