# Supplementary material for: Pou5f1/Oct4 Promotes Cell Survival via Direct Activation of mych Expression during Zebrafish Gastrulation
Source: PLoS One. 2014 Mar 18;9(3):e92356. doi: 10.1371/journal.pone.0092356 (PMC3958507; doi:10.1371/journal.pone.0092356)
Supplement: Table S2 — Analysis of the Mych contribution to the morphology of the MZ spg mutant phenotype. (Referring to: Figure 5) (PDF) [file pone.0092356.s009.pdf]

Supplemental Table S2

Referring to: Figure 5
Analysis of the Mych contribution to the morphology of the MZ*spg* mutant phenotype

		**WT**				**MZspg**	
		4.6 ng		1.4 ng		112 pg	
		***mych*-SP-MO**	**CO**	***mych*-ATG-MO**	**CO**	***mych* mRNA**	***gfp* mRNA**
**Number of Embryos**	dead by 90% epiboly	3	2	16	19	0	0
	phenotype at 25 hpf: WT	56	66	68	56	-	-
	phenotype at 25 hpf: MZ*spg*	-	-	-	-	94	92
	embryos analyzed	59	68	84	75	94	92
