# Supplementary material for: Pou5f1/Oct4 Promotes Cell Survival via Direct Activation of mych Expression during Zebrafish Gastrulation
Source: PLoS One. 2014 Mar 18;9(3):e92356. doi: 10.1371/journal.pone.0092356 (PMC3958507; doi:10.1371/journal.pone.0092356)
Supplement: Table S3 — Analysis of the mitotic index at 90%-epiboly by Sytox nuclear stain. (Referring to: Figure S5) (PDF) [file pone.0092356.s010.pdf]

Supplemental Table S3 (Referring to: Figure S5)
Analysis of the mitotic index at 90%-epiboly by Sytox nuclear stain

Data presented in Fig. S5											
		**WT**		**MZ*spg***							
105 pg	***mych* mRNA**	**-**		**-**		**+**		**-**		**+**	
4.2 ng	**p53 MO**	**-**		**-**		**-**		**+**		**+**	
		**MN**	**TN**	**MN**	**TN**	**MN**	**TN**	**MN**	**TN**	**MN**	**TN**
**ID No. of Embryo**	1	28	586	24	523	43	607	55	593	18	583
	2	23	539	23	474	17	429	22	425	22	653
	3	30	371	38	788	30	533	19	408	22	406
	4	12	268	38	647	34	732	46	776	29	458
	5	9	189	42	1083	36	654	34	676	39	583
	mean:	*20.4*	*390.6*	*33.0*	*703.0*	*32.0*	*591.0*	*35.2*	*575.6*	*26.0*	*536.6*
	SEM:	*4.23*	*76.24*	*3.95*	*109.45*	*4.30*	*51.82*	*6.88*	*71.17*	*3.70*	*45.33*
	mitotic index:	0.052227343		0.046941679		0.054145516		0.061153579		0.048453224	
	error:	0.014865771		0.009218517		0.008689576		0.014144406		0.008020628	

Additional parallel experiment											
		**WT**		**MZ*spg***							
105 pg	***mych* mRNA**	**-**		**-**		**+**		**-**		**+**	
4.2 ng	**p53 MO**	**-**		**-**		**-**		**+**		**+**	
		**MN**	**TN**	**MN**	**TN**	**MN**	**TN**	**MN**	**TN**	**MN**	**TN**
**ID No. of Embryo**	1	27	477	16	372	12	351	16	458	25	472
	2	11	260	4	324	20	427	12	413	25	372
	3	22	571	13	349	26	447	17	443	16	377
	4	22	835	11	364	22	300	17	493	32	339
	5	16	481	8	252	9	377	14	380	17	369
	mean:	*19.6*	*524.8*	*10.4*	*332.2*	*17.8*	*380.4*	*15.2*	*437.4*	*23.0*	*385.8*
	SEM:	*2.77*	*92.92*	*2.06*	*21.65*	*3.17*	*26.41*	*0.97*	*19.28*	*2.95*	*22.55*
	mitotic index:	0.037347561		0.031306442		0.04679285		0.0347508		0.059616382	
	error:	0.008458114		0.006539562		0.008940634		0.002694527		0.008402056	

MN: number of mitotic nuclei
TN: total number of nuclei
