# Supplementary material for: Pou5f1/Oct4 Promotes Cell Survival via Direct Activation of mych Expression during Zebrafish Gastrulation
Source: PLoS One. 2014 Mar 18;9(3):e92356. doi: 10.1371/journal.pone.0092356 (PMC3958507; doi:10.1371/journal.pone.0092356)
Supplement: Table S4 — Mych overexpression and p53 knockdown suppress cell death in MZ spg gastrulae. (Referring to: Figure 7) (PDF) [file pone.0092356.s011.pdf]

Supplemental Table S4

Referring to: Figure 7
Mych overexpression and p53 knockdown suppress cell death in MZ*spg* gastrulae

1st experiment (Fig. 7)						
		**WT**	**MZ*spg***			
105 pg	***mych* mRNA**	**-**	**-**	**+**	**-**	**+**
4.2 ng	**p53 MO**	**-**	**-**	**-**	**+**	**+**
		**Number of TUNEL stained cells (identified by Volocity)**				
**ID No. of Embryo**	1	2	272	42	78	2
	2	2	320	21	122	0
	3	1	354	193	95	0
	4	1	275	41	289	1
	5	13	210	52	95	6
	6	3	527	58	250	
	7		288	27	127	
	8			62	221	
	9			48		
	mean:	*3.7*	*320.9*	*60.4*	*159.6*	*1.8*
	SEM:	*1.89*	*38.25*	*17.16*	*28.71*	*1.11*
t-test	MZ*spg* : (control) vs. (*mych* mRNA inj.): P=0.0002<br>MZ*spg* : (mych *mRNA* inj) vs. (*mych* mRNA + p53MO inj.: P=0,0091					

2nd experiment (additional data)						
		**WT**	**MZ*spg***			
79 pg	***mych* mRNA**	**-**	**-**	**+**	**-**	**+**
4.2 ng	**p53 MO**	**-**	**-**	**-**	**+**	**+**
		**Number of TUNEL stained cells (identified by Volocity)**				
**ID No. of Embryo**	1	0	262	170	80	9
	2	3	235	257	290	5
	3	3	261	127	78	20
	4	1	273	124	156	4
	5	0	222	191	290	55
	6	0	278	168	257	22
	7	9	200	160	124	0
	8	0	191	157	177	1
	9	18	133	239	129	22
	10	5	420	117	157	1
	11	1	348	173	74	55
	12	8	222		179	6
	13	3				0
	14	0				8
	Mean:	*3.6*	*253.8*	*171.2*	*165.9*	*14.9*
	SEM:	*1.36*	*21.56*	*13.42*	*22.40*	*5.00*
t-test	MZ*spg:* (control) vs. (*mych* mRNA inj.): P=0.0044<br>*MZspg*: (*mych* mRNA inj) vs. (*mych* mRNA + p53MO inj.): P=0,00000008					
